# T-cell-dependent mechanisms promote Ebola VLP-induced antibody responses, but are dispensable for vaccine-mediated protection

**DOI:** 10.1038/emi.2017.31

**Published:** 2017-06-07

**Authors:** Christopher L Cooper, Karen A Martins, Sabrina M Stronsky, David P Langan, Jesse Steffens, Sean Van Tongeren, Sina Bavari

**Affiliations:** 1 Molecular and Translational Sciences, United States Army Medical Research Institute of Infectious Diseases, Frederick, MD 21702, USA; 2 Molecular Microbiology and Immunology, University of Maryland School of Medicine, Baltimore, MD 21201, USA

**Keywords:** adjuvant, Ebola, germinal center B-cell, T-cell dependent antibody, vaccine, virus-like particle

## Abstract

Humoral responses are essential for the protective efficacy of most Ebola virus (EBOV) candidate vaccines; however, the *in vivo* development of protective anti-EBOV B-cell responses is poorly defined. Here, by using the virus-like particle (VLP) as a model antigen, we demonstrate that humoral responses are generated through follicular B-cell and T-cell-dependent mechanisms in a mouse model of EBOV infection. In addition, we show that the inclusion of the clinical-grade dsRNA adjuvant known as poly-ICLC in VLP vaccinations both augments and sustains germinal center B-cell reactions, antigen-specific B-cell frequencies and anti-EBOV serum titers. Finally, we used mice that were deficient in either B-cells or T-cell-dependent antibody production to distinguish the contributing roles of EBOV humoral responses. We demonstrate that while anti-EBOV antibody responses promote protection, VLP-vaccinated mice can survive EBOV infection in the absence of detectable anti-EBOV antibodies. Moreover, we found that adjuvant signaling could circumvent the complete requirement for B-cell immunity in protection against EBOV. Collectively, these studies may prove valuable for the characterization and future development of additional EBOV vaccine candidates.

## INTRODUCTION

The 2013–2016 Ebola virus disease (EVD) epidemic in Western Africa, which involved more than 28 000 individuals and claimed more than 11 000 lives (World Health Organization, http://www.who.int/en/; Centers for Disease Control and Prevention, http://www.cdc.gov/), triggered the advancement of several medical countermeasures against the Ebola virus (EBOV), some of which had been in development for decades.^1, 2, 3^ Multiple vaccine platforms expressing or consisting of the EBOV trimeric glycoprotein (GP_1,2_) have been generated, and several are currently under clinical evaluation.^4, 5, 6, 7, 8^ However, there are fundamental gaps in our understanding of how candidate vaccines induce protective host responses, and a definitive immune correlate for vaccine efficacy has yet to be defined.^9, 10^

On the basis of studies using nonhuman primate (NHP) and rodent models of EVD, achieving both cellular and humoral responses against EBOV is thought to be critical for protection.^11, 12^ The roles of EBOV-specific CD4^+^ and CD8^+^ T-cell responses have been extensively characterized; nonetheless, they remain complex and appear to be largely vaccine platform-dependent. Adenovirus 5 and Venezuelan equine encephalitis virus-like replicon-based EBOV vaccine studies have demonstrated a critical role for CD8^+^ T-cell responses in protecting against NHP and murine EBOV infection.^13, 14, 15^ However, dispensable roles for EBOV-specific CD8^+^ T-cell responses have been supported by studies using recombinant vesicular stomatitis virus and adenovirus Hu5 vaccine platforms.^16, 17, 18^ Murine studies from our group that used EBOV viral-like particles (VLPs) have shown a similar importance for T-cells; however, adjuvants have impacted the establishment and relative contributions of these responses in protecting against EVD.^19, 20, 21^ Despite reported differences and alternative mechanisms for T-cell-mediated immunity between EBOV-based vaccine platforms, most converge on an obligate requirement for humoral responses.^16, 17, 18, 21, 22^ Further supporting this importance is the preclinical success of anti-EBOV antibodies (for example, ZMapp, ZMab) in treating EBOV infection.^23, 24, 25^

The production of antibodies following a direct interaction between B-cells and the cognate antigen can occur through T-cell-independent mechanisms (TI); however, the generation of high-affinity class-switched antibodies is dependent on follicular T-cell help (T_FH_). The formation of T-cell-dependent (TD) antibody responses can be shaped through several key events including (i) the induction of germinal center (GC) B-cell reactions; (ii) T_FH_ quantity and quality; (iii) activation-induced cytidine deaminase (AICDA/AID)-mediated somatic hypermutation and isotype class-switching; and (iv) the selection and differentiation of GC B-cells to form the antigen-specific B-cell compartment.^26, 27^ However, *in vivo* studies that define how EBOV B-cell immunity is established, the relative contributions of TI and TD B-cell mechanisms and the direct requirement for B-cell responses to protect against EBOV infection have been limited to date.^17, 28^

Previously, we demonstrated that the vaccination of laboratory mice, guinea pigs, or NHPs with a VLP consisting of EBOV matrix protein (VP-40) and GP_1,2_ elicited complete protection against EBOV infection.^29, 30, 31^ We also discovered that the inclusion of the clinical-grade dsRNA polyinosinic-polycytidylic acid (poly-IC) derivative known as poly-IC poly-l-lysine carboxymethylcellulose (poly-ICLC) in VLP vaccine preparations increased EBOV GP_1,2_-specific antibody titers and durable protection from EVD in mice.^19, 20^ Here, we use VLP as a model system to examine the establishment and requirement for EBOV B-cell immunity in mice and the impact of poly-ICLC adjuvant signaling on VLP-mediated B-cell responses.

## MATERIALS AND METHODS

### Reagents

Poly-ICLC (Hiltonol) was provided by Oncovir (Washington, DC, USA). Ebola VLPs were produced as previously described.^20, 30^ In brief, 293T cells were transfected with Ebola Zaire (Kikwit) virus glycoprotein and VP-40. VLP supernatants were collect at 72 h post-transfection, they were sucrose gradient-purified, and their total protein content was determined using a bicinchoninic acid protein assay. To ensure sterility, the VLPs were irradiated at 1e6 rad, and they contained less than 25 EU/mL endotoxin and less than 10 colony-forming units (CFU) of bacteria per vaccination. The particular lot of VLP within this study was previously used for EBOV vaccine studies and has been extensively characterized.^19, 32^ The GP content for these studies was determined by Western blot and fixed at a 10 μg GP dose for the vaccinations. The VLPs were maintained at −80 °C and diluted in sterile saline and/or combined with poly-ICLC prior to vaccination.

### Mouse strain and vaccinations

C57BL/6 (NCI Charles River Strain Code 027, Jackson Stock No. 000664), CD40-deficient (Jackson Stock No. 002928), μMT (Jackson Stock No. 002288) and AID-deficient mice (kind gifts from Drs Pat Gearhart, Robert Maul, NIAIA, Baltimore and Rafael Casellas, NIH, Bethesda) were each vaccinated intramuscularly with VLP (10 μg GP_1,2_ content) or VLP (10 μg GP_1,2_ content) plus 10 μg of poly-ICLC at 3-week intervals (day 0, day 21).

### Enzyme-linked immunosorbent assays

ELISAs were performed as previously described.^19^ In brief, blood was collected from the vaccinated mice at the indicated time points in Vacutainer serum-separating tubes. ELISA plates were coated with recombinant mammalian cell-expressed EBOV GP_1,2_ at 2  μg/mL in phosphate-buffered saline (PBS; Corning Life Sciences, Corning, NY, USA). The sera were diluted by half-log dilutions starting at 1:100, and then incubated for 1 h on GP_1,2_-coated plates. The plates were washed and then incubated with the indicated secondary horseradish peroxidase (HRP) antibody. ELISAs were developed using 3,3′,5,5′-tetramethylbenzidine (TMB) substrate/stop solution and measured on a Tecan plate reader. The absorbance cut-off was determined as the background+0.2 O.D.

### Flow cytometry

Single-cell suspensions of draining lymph nodes and spleens were collected at the indicated time points. The cells were washed using FACS buffer (PBS, 0.5% BSA and 2 mM EDTA; Corning, Sigma, St Louis, MO, USA), lysed with red blood cell (RBC) buffer (Sigma), and subsequently counter-stained. The B-cell staining included B220 (Becton-Dickinson Biosciences (BD), Franklin Lanes, NJ, USA Clone RA3-6B2), IgM (BD Clone R6-60.2), IgD (eBioscience, San Diego, CA, USA Clone 11-26C), CD38 (BD Clone 90), CD95 (BD Clone Jo-2), and T & B Cell Activation Antigen (BD Clone GL-7). T follicular helper cell staining was performed by a primary incubation with CXCR5 (BD Clone 2G8) followed by secondary incubation using goat anti-rat (H+L)-biotin (Jackson ImmunoResearch, West Grove, PA, USA 112-067-003). Subsequently, the cells were counter-stained with streptavidin (BD 557598), CD3 (BD Clone 500A2), CD4 (BD Clone RM4-5), PD-1 (eBioscience Clone RMP1-30), and ICOS (BD Clone 7E.17G9). All the samples were Fc-blocked (anti-CD16/CD32, BD) and stained to evaluate their viability (live/dead aqua, Invitrogen, Carlsbad, CA, USA) before counter-staining. The data were collected on a BD FACSCanto II or BD FACSAria II and analyzed using FlowJo (Treestar, Ashland, OR, USA).

### Neutralization assays

Neutralizing antibody titers from the serum samples were determined using recombinant vesicular stomatitis Indiana virus (rVSV) particles that coexpressed EBOV GP_1,2_ and enhanced green fluorescent protein (eGFP) (a kind gift from Kartik Chandran, Albert Einstein College of Medicine). In brief, serum dilutions beginning at 1:10 were incubated at 37 °C for 1 h with rVSV and 5% v/v Hemo-lo guinea pig complement (Cedarlane Laboratories, Burlington, NC, USA). rVSV/serum complexes were then incubated with 1 × 10^5^ FreeStyle 293F cells (ThermoFisher Scientific, Waltham, MA, USA) at 1 × 10^6^ cells/mL for 18–20 h at 37 °C. The infection percentage, as determined by eGFP expression, was measured using fluorescence-activated cell sorting (FACS). The data were collected on a BD Canto II and LSR II. The neutralization was calculated by normalizing the infection percentages to the infections performed in the presence of control sera from non-vaccinated laboratory mice.

### B-cell ELISPOTs

ELISPOTs were performed according to the MabTech (Cincinnati, OH, USA) protocol. In brief, cryopreserved splenocyte populations were thawed, washed 2 × with complete medium, and adjusted to 2 × 10^6^ cells/mL. Splenocyte suspensions (0.1 mL) were added to recombinant EBOV GP_1,2_-coated ELISPOT plates and then incubated overnight at 37 °C with 95/5 oxygen/CO_2_. The plates were then washed, incubated with biotin-IgG secondary antibody (MabTech) followed by streptavidin-HRP (MabTech) and developed using TMB substrate/stop solution. Imaging/counting was performed using a CTL Immunospot instrument (Cellular Technology, Shaker Heights, OH, USA).

### Ebola virus infections

Laboratory mice were inoculated intraperitoneally with a target dose of 1000 pfu of mouse-adapted Ebola virus/*H. sapiens*-tc/COD/1976/Yambuku-Mayinga (ma-EBOV) on day 28 after the last vaccination or during secondary inoculations. All the live-virus studies were conducted under maximum (biosafety level 4) containment. Clinical observations were recorded throughout the study starting on the day of virus inoculation. Moribund mice were euthanized according to institution-approved clinical scoring.

### Statistical analysis

All the statistical analyses were performed using GraphPad Prism (Windows V6). Data comparisons using unpaired parametric Student’s *t*-tests were performed for titer analysis and immune populations. A survival curve comparison was performed using a log-rank (Mantel–Cox) test.

## RESULTS

### VLP vaccination induces robust germinal center B-cell responses, which are augmented with the inclusion of poly-ICLC

**Figure 1:**
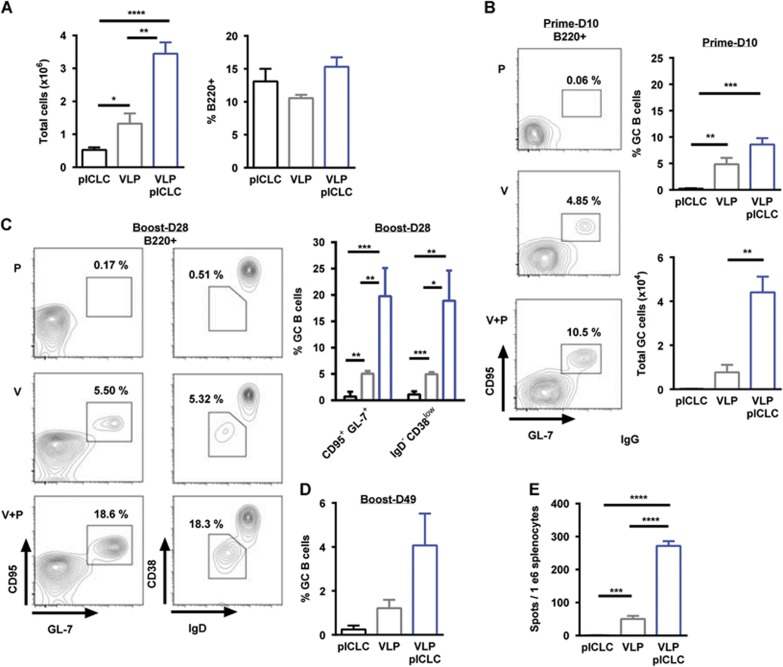
Poly-ICLC augments and sustains VLP-mediated germinal centers. Mice were vaccinated with VLP or VLP plus poly-ICLC (pICLC), and their draining lymph nodes (dLNs) were isolated. Single-cell suspensions were stained with B220, IgD, IgM, CD38, CD95, GL-7 and live/dead dye and collected by FACS. (**A**) Left, day 10 dLN cellularity; right, relative percentage of the B220^+^ population on day 10 dLN. (**B**) A representative FACS plot of the relative percentage and total number of day 10 dLN B220^+^CD95^+^GL7^+^ GC B-cells. (**C**) A representative day 28 (day 7 post-boosting) FACS plot and the relative frequency of B220^+^CD95^+^GL7^+^ GC B-cells (left panels and top right) and of B220^+^CD38^lo^IgD^−^IgM^−^ B-cells (right panels and top right). (**D**) The relative percentage of day 49 (day 28 post-boosting) dLN B220^+^CD95^+^GL7^+^ GC B-cells. (**E**) The relative frequency of day 25 (day 4 post-boosting) EBOV GP_1,2_-specific B-cells in the spleen as measured by ELISPOT. For clarity in the graphics, only significant comparisons have been labeled (means, s.e.m., *n*=3–5; *P<0.05, **P<0.005, and ***P<0.0005). All the other statistical comparisons are assumed to be non-significant.

Prior VLP vaccination studies have demonstrated the induction of EBOV GP_1,2,_ antibody responses and that these responses can be heightened with the inclusion of adjuvants. Recently, we demonstrated that the dsRNA adjuvant poly-ICLC provided enhanced VLP-mediated anti-EBOV titers and influenced the antibody-isotype.^19, 20^ The production of high-affinity class-switched antibodies is dependent on germinal center (GC) formation;^26, 33^ however, limited characterization of this B-cell compartment has been reported for vaccine platforms that were developed against EBOV.^28^ Therefore, we examined if VLP vaccination resulted in the generation of GC reactions and if poly-ICLC would impact these responses. Mice were vaccinated with VLP (intramuscularly) in the presence or absence of poly-ICLC using a prime-boost schedule at three-week intervals (day 0, day 21). VLP-vaccinated mice were characterized by an approximate three-fold increase in draining lymph node (dLN, popliteal) cellularity at day 10 subsequent to prime vaccination, whereas the inclusion of poly-ICLC resulted in an approximate seven-fold increase (Figure 1A). However, the relative frequency of B220^+^ B-cells was unaltered (Figure 1A). On day 10, subsequent to prime vaccination, GC B-cells (B220^+^GL7^+^CD95^+^) were clearly identified within the dLN of both VLP-vaccinated groups. However, the relative frequencies and total numbers of GC B-cells were increased in the presence of poly-ICLC (Figure 1B).

We next determined the impact of vaccine boosting on VLP-mediated GC reactions. As with prime vaccination, GC B-cells were detected within the dLN on day 28 (day 7 post-boosting), and, consistent with the prime vaccination results, the inclusion of the adjuvant significantly augmented the relative frequency of GC B-cells (Figure 1C, left panel). Additional phenotypic characterization of the B-cell compartment supported the induction of activated class-switched (B220^+^GL7^+^CD95^+^CD38^low^IgD^−^IgM^−^) B-cells following VLP vaccination with heightened frequencies observed when adding poly-ICLC (Figure 1C, right panel). Notably, the relative frequency of GC B-cells increased after boosting in the presence of poly-ICLC, whereas VLP-only-vaccinated mice presented comparable GC B-cell frequencies following both prime and boost vaccinations (Figures 1B and 1C). VLP-mediated GC reactions were still present within the dLN at four weeks post-boosting (day 49) with a continued increase in GC B-cell frequencies being observed in the presence of the adjuvant (Figure 1D). Consistent with GC B-cell dynamics, the VLP-induced antibody responses plateaued following vaccination in the absence of the adjuvant. However, the inclusion of poly-ICLC resulted in a continual rise of total anti-GP_1,2_ IgG titers and enhanced EBOV neutralization up to one month post-final vaccination (day 49), a time-point associated with acute protection from EBOV infection (Supplementary Figures S1A and S1C). Durable (>5 months post-vaccination) anti-EBOV GP_1,2_ IgG responses were additionally augmented in the presence of poly-ICLC (Supplementary Figure S1B, top). Interestingly, after one, two or three vaccinations, the EBOV GP_1,2_-specific IgM responses were marginal and were detected only in the presence of the adjuvant (Supplementary Figure S1B, bottom).

Because productive GC reactions result in the generation of antibody-secreting cells (ASCs), we measured the frequency of EBOV GP_1,2_-specific B-cells. Antigen-specific B-cells could not be detected after prime vaccination (data not shown), but we observed an approximate five-fold increase in ASC frequencies after the inclusion of poly-ICLC following boosting (Figure 1E). GP_1,2_-specific B-cells were still detectable up to day 49 in the presence of adjuvant, but they approached our lower limit of detection for VLP vaccination alone (data not shown). Altogether, we demonstrate that VLP-mediated B-cell responses are associated with GC formation and that the addition of poly-ICLC as an adjuvant resulted in augmented GC B-cell frequencies, increased generation of EBOV GP_1,2_-specific ASCs and elevated antibody titers.

### VLP-mediated humoral immunity is established through follicular B-cell and T-cell-dependent mechanisms, but it is partially dispensable for protection

**Figure 2:**
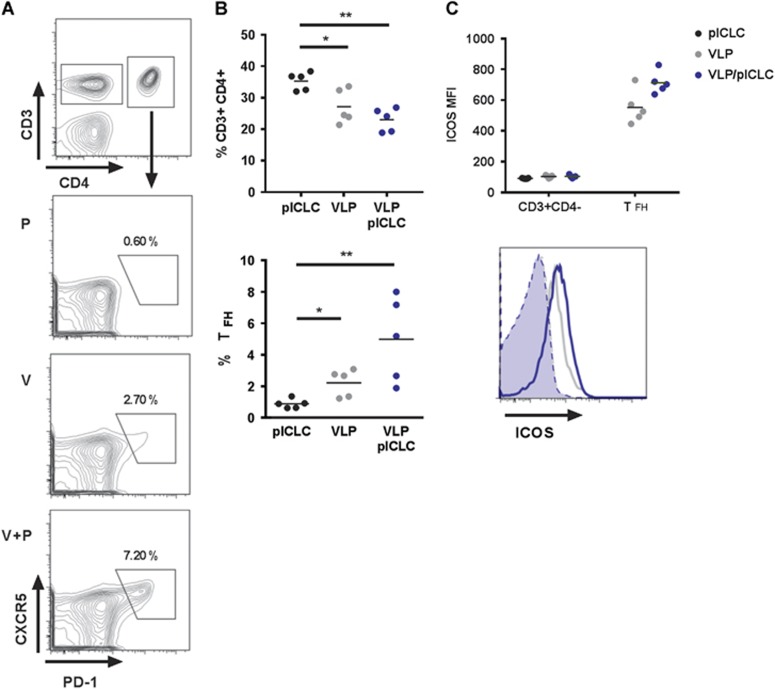
VLP-mediated humoral responses are dependent on follicular T-cell help. Mice were vaccinated with VLP or VLP plus poly-ICLC. Single-cell suspensions from the draining lymph nodes were stained with CD4, CD3, CXCR5, PD-1, ICOS, and live/dead dye and then analyzed by FACS. (**A**) The representative gating of day 7 prime T_FH_ subsets. (**B**) Top, relative percentage of CD3^+^CD4^+^ and bottom, T_FH_ frequency. (**C**) Top, ICOS mean fluorescent intensity of respective T-cell subsets subsequent to different vaccination regimes; Bottom, representative ICOS surface expression of CD3^+^CD4^−^ (shaded) and CD3^+^CD4^+^PD-1^+^CXCR5^hi^ (open) T_FH_ cells. For graphic clarity, only the significant comparisons have been labeled (means, s.e.m.; *n*=5, *P<0.05, **P<0.005). All the other statistical comparisons are assumed to be non-significant.

The quality and quantity of GC B-cell formation is dependent on T_FH_ cells.^34^ Consistent with our recent findings and the relationship between T_FH_ and the formation of GC reactions,^19, 35^ VLP-mediated T_FH_ (CD3^+^CD4^+^PD-1^+^CXCR5^hi^) cells are generated during vaccination (Figures 2A and 2B). Moreover, the relative size of the T_FH_ compartment is increased with the addition of adjuvant, suggesting that poly-ICLC augments humoral immunity by promoting T_FH_ and GC B-cell reactions (Figures 2A and 2B). Consistent with the cellular phenotyping, high levels of inducible T-cell costimulator (ICOS, CD278) was detected on T_FH_ cells following vaccination with a subtle increase of expression after adjuvant inclusion (Figure 2C). Interestingly, the relative frequency of dLN CD3^+^CD4^+^ T-cells declined subsequent to vaccination (Figure 2B).

**Figure 3:**
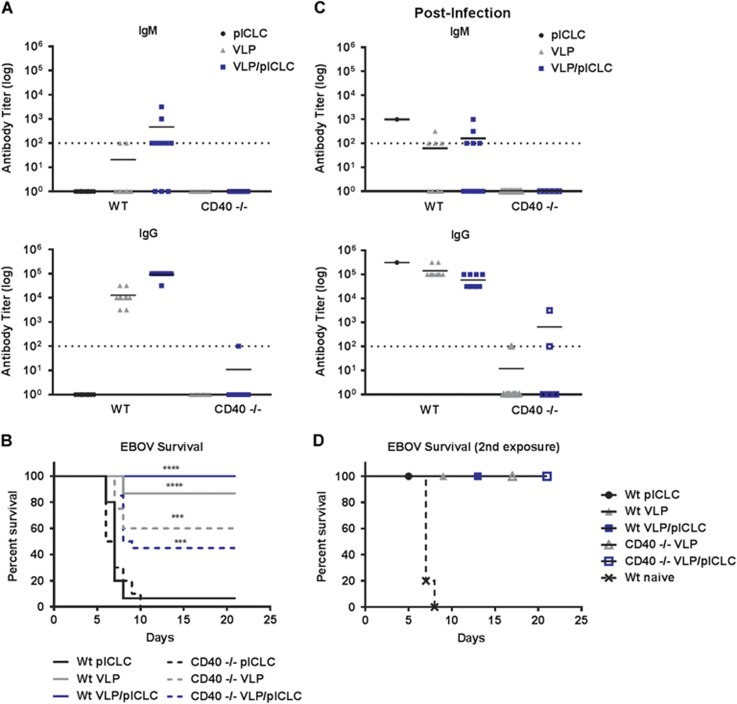
EBOV GP antibody responses are established through T-cell-dependent mechanisms. (**A**) Wild-type or CD40^−/−^ mice were vaccinated with VLP with or without poly-ICLC at day 0 and day 21. Their sera were collected at day 35 (day 14 post-boosting) and EBOV GP_1,2_-specific IgG and IgM responses were measured by ELISA. (**B**) On day 49 (four weeks post-vaccination), wild-type or CD40^−/−^ VLP-vaccinated mice were inoculated with a typically lethal dose of EBOV. The combined survival from two independent vaccination studies (*n*=15/group for wild-type mice; *n*=20/group for CD40^−/−^-vaccinated mice; and *n*=10/group CD40^−/−^ control). The control wild-type and CD40^−/−^ mice displayed similar lethality in response to ma-EBOV with mean survivals of 7 and 6.5 days, respectively; *P*=0.62. ***P<0.0005, ****P<0.0001. (**C**) Sera were collected from the surviving mice on day 21 post-EBOV infection, and their GP_1,2_-specific IgG and IgM responses were measured by ELISA. (**D**) The naïve or vaccinated mice that survived the initial EBOV infection were once again inoculated with a typically lethal dose of EBOV on day 28 after initial infection and monitored for survival. All titers were calculated by reciprocal end-point dilutions with the background set at the control absorbance +0.2 O.D. Dashed lines represent the levels of detection.

CD40-CD40L interactions between GC B-cells and T_FH_ cells are required to induce productive T-cell-dependent (TD) antibody responses.^36^ We therefore determined the direct *in vivo* contribution of this pathway during VLP vaccination. The tumor necrosis factor superfamily, receptor 5 knockout (CD40^−/−^) mice have defective TD humoral immune responses while also being capable of establishing TI antibody responses.^37^ Wild-type and CD40^−/−^ mice were prime-boost-vaccinated (day 0, day 21) with VLP and EBOV GP_1,2_-specific antibody titers were analyzed on day 35. Wild-type mice that were vaccinated with VLP displayed robust IgG antibody titers; however, the CD40^−/−^ mice had no detectable anti-GP_1,2_-specific IgG or IgM responses (Figure 3A and Supplementary Figure S2A). The VLP candidate vaccine consists of both EBOV GP and VP-40; we therefore also determined antibody reactivity against VP-40. Consistent with the lack of GP_1,2_ antibody responses, the CD40^−/−^ VLP-vaccinated mice also failed to establish anti-VP-40 humoral responses (Supplementary Figure S2B).

Previous *in vivo* characterizations of EBOV candidate vaccines have suggested an obligate requirement for anti-EBOV antibodies for efficacy.^16, 17, 21, 22^ We therefore tested the protective contribution of humoral immunity using CD40^−/−^ mice, which displayed a lack of VLP-specific antibody responses. The prime-boost vaccination of wild-type mice with VLP or VLP/poly-ICLC resulted in acute protection against a typically lethal dose of EBOV (Figure 3B). Surprisingly, we also observed partial protection in VLP-vaccinated CD40^−/−^ mice (Figure 3B). In two separate studies, the control mice succumbed to EBOV infection by day 10, whereas a combined 60% (*n*=24/40) of the VLP-vaccinated mice were protected from disease in the absence of detectable EBOV GP_1,2_-specific antibody responses. Importantly, we observed similar morbidity within unvaccinated wild-type (mean survival= 7 days) and CD40^−/−^ mice (mean survival=6.5 days; *P*-value= 0.62), suggesting comparable EBOV lethality across murine strains. Poly-ICLC did not significantly alter the protection in VLP-vaccinated CD40^−/−^ mice (Figure 3B).

We speculated that despite the lack of TI responses upon VLP vaccination, EBOV infection may result in the generation of protective low-affinity antibody responses. To test this hypothesis, we analyzed the antibody responses following EBOV infection in the mice that had survived EBOV infection. All the surviving wild-type mice had robust IgG titers against EBOV GP_1,2_, whereas the CD40^−/−^ mice had a near complete absence of antibody responses, even subsequent to EBOV infection (Figure 3C). Of the 14 CD40^−/−^ survivors analyzed after EBOV infection, only 3 had marginal anti-EBOV GP_1,2_ IgG titers and none mounted detectable IgM responses (Figure 3C). On day 28 subsequent to primary EBOV infection, the mice were once again inoculated with a typically lethal dose of EBOV. Consistent with the initial survival results, all the mice were protected from secondary EBOV infection, including those mice without detectable anti-GP_1,2_ antibody responses (Figure 3D). Altogether, these results support the idea that EBOV-specific antibody responses following either VLP vaccination or EBOV infection are generated through follicular B-cell and TD mechanisms. However, protection from EBOV lethality could be achieved in the absence of these TD humoral immune responses.

### Protection from EBOV infection in the absence of antibody affinity maturation or class-switched humoral responses

**Figure 4:**
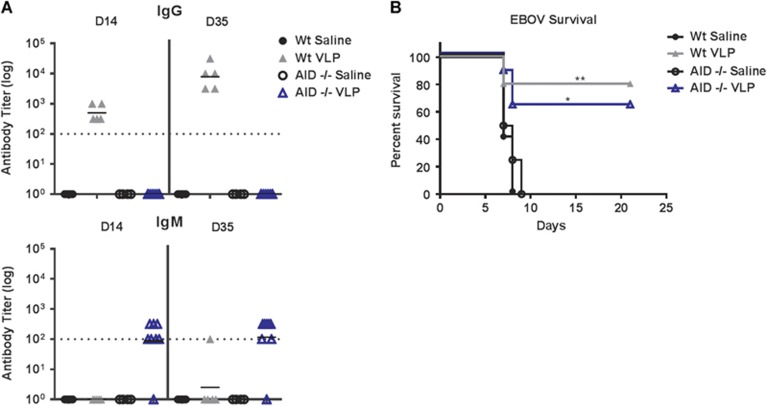
Protection from EBOV lethality without high-affinity or class-switched GP-specific antibodies. (**A**) Wild-type or AID^−/−^ mice were vaccinated with VLP on day 0 and day 21. Their sera were collected on day 14 and day 35 (day 14 post-boosting), and their EBOV GP_1,2_-specific IgG and IgM responses were measured by ELISA. The titers were calculated by reciprocal end-point dilutions with the background set at the control absorbance +0.2 O.D. Dashed lines represent the levels of detection. (**B**) The survival of mice that were inoculated with a typically lethal dose of EBOV on day 49 (*n*=5 wild-type mice/group; *n*=8 AID^−/−^ VLP-vaccinated mice, and *n*=4 AID^−/−^ control). The control wild-type and AID^−/−^ mice displayed similar lethality in response to ma-EBOV with mean survivals of 7 and 7.5 days, respectively; *P*=0.44. *P<0.05, **P<0.005.

Our finding in which mice survived a typical lethal EBOV inoculation in the absence of anti-EBOV GP_1,2_ antibodies is contrary to previous reports. To confirm our results, we used activation-induced cytidine deaminase-deficient (AICDA/AID^−/−^) mice that fail to generate high-affinity IgG isotype antibodies.^38^ Wild-type or AID^−/−^ mice were prime-boost-vaccinated with VLP as described above. VLP vaccination of wild-type mice generated robust IgG responses; however, AID^−/−^ mice were characterized by a complete absence of class-switched EBOV GP_1,2_-specific IgG titers (Figure 4A). To determine the protective role of these IgG responses, wild-type and AID^−/−^ mice were inoculated with a typically lethal dose of EBOV. In accordance with our CD40^−/−^ studies, we observed ~60% protection against EBOV infection in mice lacking IgG antibody responses (Figure 4B). Moreover, wild-type and AID^−/−^ control mice succumbed to EBOV infection at similar rates (wild-type mean survival=7 days, AID^−/−^ mean survival=7.5 days; *P*=0.44). VLP-vaccinated AID^−/−^ mice mounted marginal IgM responses that were not seen in wild-type mice; these responses do not appear to be required for protection (Figure 4A).

### Adjuvant-enhanced protection against EBOV infection in the complete absence of B-cells

**Figure 5:**
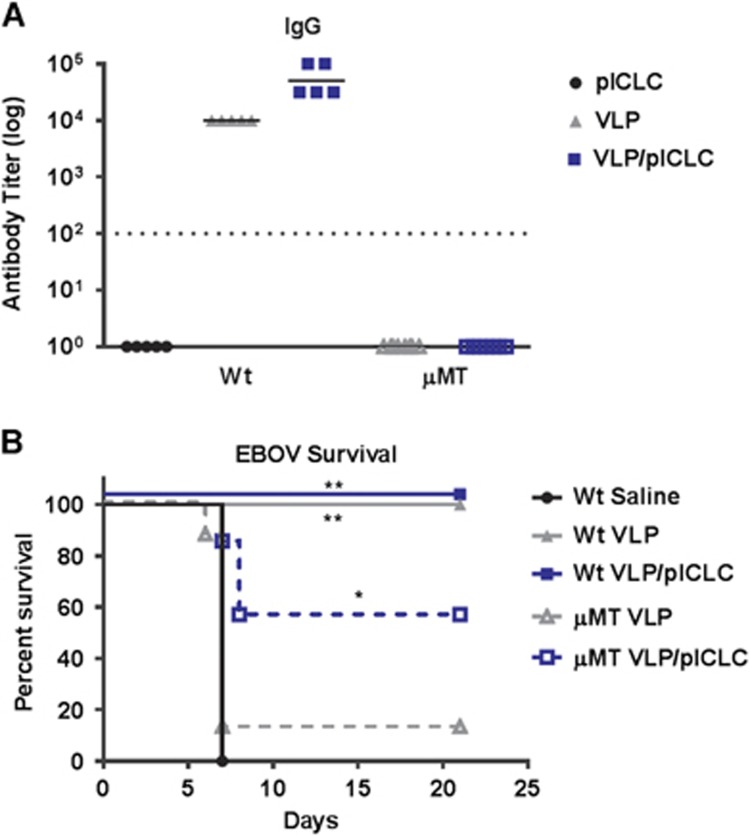
Poly-ICLC rescues VLP efficacy in B-cell-deficient animals. (**A**) Wild-type or μMT mice were vaccinated on day 0 and day 21 with or without poly-ICLC. Their sera were collected on day 35 (day 14 post-boosting), and EBOV GP_1,2_-specific IgG responses were measured by ELISA. The titers were calculated by reciprocal end-point dilutions with the background set at the control absorbance +0.2 O.D. Dashed lines represent the levels of detection. (**B**) The survival of mice that were inoculated with a typically lethal dose of EBOV on day 49 (*n*=5 wild-type mice; *n*=8 μMT VLP, and *n*=7 μMT poly/VLP). *P<0.05, **P<0.005.

Despite the agreement of our CD40^−/−^ and AID^−/−^ mouse study results, previous experiments utilizing B-cell-deficient mice (for example, μMT, Jh^−/−^) have suggested that vaccine-induced antibody responses are obligatory for protection against EBOV.^12, 17, 21^ However, μMT and Jh^−/−^ mice have a developmental block in B-cell lymphopoiesis and therefore display a complete loss of the B-cell compartment.^39^ Indeed, we observed that VLP-vaccinated μMT mice failed to establish EBOV GP_1,2_-specific antibody responses and were not protected from EBOV infection (Figures 5A and 5B). Consistent with previous reports, in the absence of B-cells, VLP vaccination alone failed to protect mice from a typically lethal dose of EBOV. However, surprisingly, the inclusion of poly-ICLC during μMT VLP vaccination rescued the protection of the mice from EBOV lethality despite the complete absence of B-cells (Figure 5B). This finding suggests that adjuvant signaling is inducing a B-cell-independent mechanism to protect against EBOV infection.

## DISCUSSION

The mechanism of protection established by EBOV vaccine platforms has been much-debated, with no definitive correlate identified to date. As EBOV candidate vaccines progress towards Food and Drug Administration (FDA) licensure in the United States, increased focus on identifying correlates of immune protection will be required. Moreover, the potential lack of human efficacy data from EBOV vaccine trials may require licensure in the United States via the FDA ‘Animal Rule’.^10, 40^ Therefore, our ability to interpret EBOV vaccine responses accurately depends upon well-defined animal models.

The ma-EBOV model has been critical for the characterization of protective Ebola vaccines and therapeutics.^41, 42^ In addition, the ability to conduct studies in the context of wild-type and knockout (for example, Jh4 ^−/−^, β2^−/−^, CD4^−/−^, CD8^−/−^, CD11^−/−^) mouse models has provided insight into the mechanisms of protection against EBOV infection.^18, 21, 43, 44^ Previous studies have demonstrated the critical importance of anti-EBOV antibody vaccine responses.^16, 17, 22^ The preclinical success of EBOV antibody therapeutics additionally supports an essential role for humoral immunity in the protection against EVD.^23, 24, 45^ However, little is known about the early EBOV B-cell initiating events that are required for the establishment of these responses.^28^

Within this study, we show that the EBOV GP_1,2_-specific humoral responses that were induced by both VLP vaccination and EBOV infection are predominately established through T-cell-dependent mechanisms. We find that VLP vaccinations generate robust and sustained GC B-cell reactions that are detectable at least four weeks post-vaccination, and these responses were augmented through dsRNA signaling as provided by poly-ICLC. Of interest, GC B-cell responses were not detectable following vaccination with equal amounts of soluble recombinant EBOV GP_1,2_, suggesting differences in GC formation associated with the display of EBOV GP_1,2_ (data not shown). Finally, our studies indicated that antibody responses promote protection against typically lethal EBOV infection, but they are neither obligate nor predictive of survival.

Our finding of protection from Ebola lethality in the absence of EBOV-specific antibodies builds upon the results of previous reports.^16, 17, 22^ Prior murine studies that defined an obligate role for antibody-mediated protection against EVD were obtained from knockout models with a complete loss of the B-cell compartment.^16, 21^ These studies demonstrated that B-cell-deficient mice were not protected following EBOV vaccination, which is consistent with our findings. Immune depletion studies within NHP models have additionally been used to delineate the requirement for B-cells in vaccine-induced EBOV immunity, and previous EBOV rVSV vaccine platform studies within NHPs demonstrated a critical role for CD4^+^ T-cells in the establishment of anti-GP_1,2_ antibody responses.^17^ Our data support a similar mechanism within mice and extend this observation by linking the requirement for CD40/CD40L interaction.

However, contrary to previous reports, our studies demonstrate that an absence of antibodies does not result in a complete loss of protection from EVD. The animal models (CD40^−/−^ and AID^−/−^) used within this study have no known defects in T or B-cell development and are fully capable of producing T-cell-independent antibodies. We observed ~60% protection against EBOV infection in both murine models following VLP vaccination, despite a failure to produce anti-EBOV GP_1,2_-specific antibodies. Moreover, in support of antibody-independent protection, we found that the inclusion of poly-ICLC in VLP preparations could partially rescue protection against EBOV lethality, even in animals which have a complete loss of B-cells. Understanding the mechanism by which this is achieved will be the focus of future studies.

Our results support the idea that anti-EBOV GP_1,2_-specific antibody responses are generated via follicular B-cell and TD mechanisms. Although we failed to detect TI antibody responses following either VLP vaccination or EBOV infection, we cannot preclude contributions from low-affinity antibodies that were unmeasurable within our studies. However, our finding of poly-ICLC-mediated protection in VLP-vaccinated B-cell-deficient mice would suggest a minor, if any, contribution by such mechanisms within our model. Previously, we demonstrated that EBOV-specific T-cell responses following VLP vaccination are essential for protection.^19, 20, 21^ Prior studies using the VLP as a pre- or post-exposure therapeutic agent have reported a role for innate immune components in protecting against EVD.^43, 46^ Therefore, these T-cell or innate immune responses may provide unknown compensatory mechanisms in protecting against EBOV lethality in the absence of anti-EBOV antibodies. Questions about the mechanisms of VLP efficacy in the absence of anti-EBOV antibodies are currently being investigated.

In opposition to an obligate effort by humoral components in protecting against EBOV lethality, we illustrate a division of labor for VLP-mediated anti-EBOV immunity. The finding of a loss of VLP-mediated efficacy in B-cell-deficient mice, yet protection without detectable anti-EBOV antibodies, potentially suggests an unappreciated role for antibody-independent B-cell mechanisms in promoting protection.^47, 48^ The additional finding that the requirement for B-cells could be bypassed with the inclusion of dsRNA signaling suggests further unknown redundancies or compensatory mechanisms in the protection against EBOV lethality. Together, the data in this work provide a comprehensive description of the establishment and requirement of VLP-mediated EBOV humoral immunity. Moreover, they highlight intriguing gaps in our knowledge of less conventional roles for B-cell immune functions.

## References

[bib1] Wong G, Kobinger GP. Backs against the wall: novel and existing strategies used during the 2014-2015 Ebola virus outbreak. Clin Microbiol Rev 2015; 28: 593–601. 25972518 10.1128/CMR.00014-15 PMC4429225

[bib2] Marzi A, Feldmann H. Ebola virus vaccines: an overview of current approaches. Expert Rev Vaccines. 2014; 13: 521–531. 24575870 10.1586/14760584.2014.885841 PMC4785864

[bib3] Cooper CL, Bavari S. A race for an Ebola vaccine: promises and obstacles. Trends Microbiol 2015; 23: 65–66. 25535021 10.1016/j.tim.2014.12.005

[bib4] De Santis O, Audran R, Pothin E et al. Safety and immunogenicity of a chimpanzee adenovirus-vectored Ebola vaccine in healthy adults: a randomised, double-blind, placebo-controlled, dose-finding, phase 1/2a study. Lancet Infect Dis 2016; 16: 311–320. 26725450 10.1016/S1473-3099(15)00486-7

[bib5] Huttner A, Dayer JA, Yerly S et al. The effect of dose on the safety and immunogenicity of the VSV Ebola candidate vaccine: a randomised double-blind, placebo-controlled phase 1/2 trial. Lancet Infect Dis 2015; 15: 1156–1166. 26248510 10.1016/S1473-3099(15)00154-1 PMC6624136

[bib6] Regules JA, Beigel JH, Paolino KM et al. A recombinant vesicular stomatitis virus ebola vaccine—preliminary report. N Engl J Med 2017; 376: 330–341. 25830322 10.1056/NEJMoa1414216 PMC5408576

[bib7] Martins KA, Jahrling PB, Bavari S et al. Ebola virus disease candidate vaccines under evaluation in clinical trials. Expert Rev Vaccines 2016; 15: 1101–1112. 27160784 10.1080/14760584.2016.1187566 PMC5026048

[bib8] Falzarano D, Geisbert TW, Feldmann H. Progress in filovirus vaccine development: evaluating the potential for clinical use. Expert Rev Vaccines 2011; 10: 63–77. 21162622 10.1586/erv.10.152 PMC3398800

[bib9] Krause PR, Bryant PR, Clark T et al. Immunology of protection from Ebola virus infection. Sci Transl Med 2015; 7: 286ps11. 10.1126/scitranslmed.aaa8202 25947159

[bib10] Sullivan NJ, Martin JE, Graham BS et al. Correlates of protective immunity for Ebola vaccines: implications for regulatory approval by the animal rule. Nat Rev Microbiol 2009; 7: 393–400. 19369954 10.1038/nrmicro2129 PMC7097244

[bib11] Bradfute SB, Bavari S. Correlates of immunity to filovirus infection. Viruses 2011; 3: 982–1000. 21994766 10.3390/v3070982 PMC3185794

[bib12] Wong G, Kobinger GP, Qiu X. Characterization of host immune responses in Ebola virus infections. Expert Rev Clin Immunol 2014; 10: 781–790. 24742338 10.1586/1744666X.2014.908705

[bib13] Sullivan NJ, Hensley L, Asiedu C et al. CD8+ cellular immunity mediates rAd5 vaccine protection against Ebola virus infection of nonhuman primates. Nat Med 2011; 17: 1128–1131. 21857654 10.1038/nm.2447

[bib14] Olinger GG, Bailey MA, Dye JM et al. Protective cytotoxic T-cell responses induced by venezuelan equine encephalitis virus replicons expressing Ebola virus proteins. J Virol. 2005; 79: 14189–14196. 16254354 10.1128/JVI.79.22.14189-14196.2005 PMC1280180

[bib15] Warfield KL, Olinger GG. Protective role of cytotoxic T lymphocytes in filovirus hemorrhagic fever. J Biomed Biotechnol 2011; 2011: 984241. 22253531 10.1155/2011/984241 PMC3255346

[bib16] Wong G, Richardson JS, Pillet S et al. Immune parameters correlate with protection against ebola virus infection in rodents and nonhuman primates. Sci Transl Med 2012; 4: 158ra46. 10.1126/scitranslmed.3004582 PMC3789651 23115355

[bib17] Marzi A, Engelmann F, Feldmann F et al. Antibodies are necessary for rVSV/ZEBOV-GP-mediated protection against lethal Ebola virus challenge in nonhuman primates. Proc Natl Acad Sci USA 2013; 110: 1893–1898. 23319647 10.1073/pnas.1209591110 PMC3562844

[bib18] Jones SM, Stroher U, Fernando L et al. Assessment of a vesicular stomatitis virus-based vaccine by use of the mouse model of Ebola virus hemorrhagic fever. J Infect Dis 2007; 196: S404–S412. 17940977 10.1086/520591

[bib19] Martins KA, Cooper CL, Stronsky SM et al. Adjuvant-enhanced CD4 T cell responses are critical to durable vaccine immunity. EBioMedicine 2016; 3: 67–78. 26870818 10.1016/j.ebiom.2015.11.041 PMC4739439

[bib20] Martins KA, Steffens JT, van Tongeren SA et al. Toll-like receptor agonist augments virus-like particle-mediated protection from Ebola virus with transient immune activation. PLoS ONE 2014; 9: e89735. 24586996 10.1371/journal.pone.0089735 PMC3933660

[bib21] Warfield KL, Olinger G, Deal EM et al. Induction of humoral and CD8+ T cell responses are required for protection against lethal Ebola virus infection. J Immunol 2005; 175: 1184–1191. 16002721 10.4049/jimmunol.175.2.1184

[bib22] Blaney JE, Marzi A, Willet M et al. Antibody quality and protection from lethal Ebola virus challenge in nonhuman primates immunized with rabies virus based bivalent vaccine. PLoS Pathog 2013; 9: e1003389. 23737747 10.1371/journal.ppat.1003389 PMC3667758

[bib23] Qiu X, Audet J, Wong G et al. Sustained protection against Ebola virus infection following treatment of infected nonhuman primates with ZMAb. Sci Rep 2013; 3: 3365. 24284388 10.1038/srep03365 PMC3842534

[bib24] Qiu X, Wong G, Audet J et al. Reversion of advanced Ebola virus disease in nonhuman primates with ZMapp. Nature 2014; 514: 47–53. 25171469 10.1038/nature13777 PMC4214273

[bib25] Pettitt J, Zeitlin L, Kim DH et al. Therapeutic intervention of Ebola virus infection in rhesus macaques with the MB-003 monoclonal antibody cocktail. Sci Transl Med 2013; 5: 199ra13. 10.1126/scitranslmed.3006608 23966302

[bib26] McHeyzer-Williams M, Okitsu S, Wang N et al. Molecular programming of B cell memory. Nat Rev Immunol 2012; 12: 24–34. 10.1038/nri3128 PMC3947622 22158414

[bib27] Mesin L, Ersching J, Victora GD. Germinal center B cell dynamics. Immunity 2016; 45: 471–482. 27653600 10.1016/j.immuni.2016.09.001 PMC5123673

[bib28] Bengtsson KL, Song H, Stertman L et al. Matrix-M adjuvant enhances antibody, cellular and protective immune responses of a Zaire Ebola/Makona virus glycoprotein (GP) nanoparticle vaccine in mice. Vaccine 2016; 34: 1927–1935. 26921779 10.1016/j.vaccine.2016.02.033

[bib29] Warfield KL, Dye JM, Wells JB et al. Homologous and heterologous protection of nonhuman primates by Ebola and Sudan virus-like particles. PLoS ONE 2015; 10: e0118881. 25793502 10.1371/journal.pone.0118881 PMC4368629

[bib30] Warfield KL, Bosio CM, Welcher BC et al. Ebola virus-like particles protect from lethal Ebola virus infection. Proc Natl Acad Sci USA 2003; 100: 15889–15894. 14673108 10.1073/pnas.2237038100 PMC307663

[bib31] Warfield KL, Swenson DL, Olinger GG et al. Ebola virus-like particle-based vaccine protects nonhuman primates against lethal Ebola virus challenge. J Infect Dis 2007; 196: S430–S437. 17940980 10.1086/520583

[bib32] Cazares LH, Ward MD, Brueggemann EE et al. Development of a liquid chromatography high resolution mass spectrometry method for the quantitation of viral envelope glycoprotein in Ebola virus-like particle vaccine preparations. Clin Proteomics 2016; 13: 18. 27597813 10.1186/s12014-016-9119-8 PMC5011338

[bib33] De Silva NS, Klein U. Dynamics of B cells in germinal centres. Nat Rev Immunol 2015; 15: 137–148. 25656706 10.1038/nri3804 PMC4399774

[bib34] Ma CS, Deenick EK, Batten M et al. The origins, function, and regulation of T follicular helper cells. J Exp Med 2012; 209: 1241–1253. 22753927 10.1084/jem.20120994 PMC3405510

[bib35] Baumjohann D, Preite S, Reboldi A et al. Persistent antigen and germinal center B cells sustain T follicular helper cell responses and phenotype. Immunity 2013; 38: 596–605. 23499493 10.1016/j.immuni.2012.11.020

[bib36] Elgueta R, Benson MJ, de Vries VC et al. Molecular mechanism and function of CD40/CD40L engagement in the immune system. Immunol Rev 2009; 229: 152–172. 19426221 10.1111/j.1600-065X.2009.00782.x PMC3826168

[bib37] Kawabe T, Naka T, Yoshida K et al. The immune responses in CD40-deficient mice: impaired immunoglobulin class switching and germinal center formation. Immunity 1994; 1: 167–178. 7534202 10.1016/1074-7613(94)90095-7

[bib38] Muramatsu M, Kinoshita K, Fagarasan S et al. Class switch recombination and hypermutation require activation-induced cytidine deaminase (AID), a potential RNA editing enzyme. Cell 2000; 102: 553–563. 11007474 10.1016/s0092-8674(00)00078-7

[bib39] Kitamura D, Roes J, Kuhn R et al. B cell-deficient mouse by targeted disruption of the membrane exon of the immunoglobulin mu chain gene. Nature 1991; 350: 423–426. 1901381 10.1038/350423a0

[bib40] Allio T. Product development under FDA's animal rule: understanding FDA's expectations and potential implications for traditional development programs. Therap Innov Regul Sci 2016; 50: 660–670. 10.1177/2168479016641717 30231765

[bib41] Bray M, Davis K, Geisbert T et al. A mouse model for evaluation of prophylaxis and therapy of Ebola hemorrhagic fever. J Infect Dis 1998; 178: 651–661. 9728532 10.1086/515386

[bib42] Nakayama E, Saijo M. Animal models for Ebola and Marburg virus infections. Front Microbiol 2013; 4: 267. 24046765 10.3389/fmicb.2013.00267 PMC3763195

[bib43] Bradfute SB, Anthony SM, Stuthman KS et al. Mechanisms of immunity in post-exposure vaccination against Ebola virus infection. PLoS ONE 2015; 10: e0118434. 25785602 10.1371/journal.pone.0118434 PMC4364937

[bib44] Bradfute SB, Warfield KL, Bray M. Mouse models for filovirus infections. Viruses 2012; 4: 1477–1508. 23170168 10.3390/v4091477 PMC3499815

[bib45] Furuyama W, Marzi A, Nanbo A et al. Discovery of an antibody for pan-ebolavirus therapy. Sci Rep 2016; 6: 20514. 26861827 10.1038/srep20514 PMC4748290

[bib46] Warfield KL, Perkins JG, Swenson DL et al. Role of natural killer cells in innate protection against lethal ebola virus infection. J Exp Med 2004; 200: 169–179. 15249592 10.1084/jem.20032141 PMC2212007

[bib47] Nanton MR, Way SS, Shlomchik MJ et al. Cutting edge: B cells are essential for protective immunity against Salmonella independent of antibody secretion. J Immunol 2012; 189: 5503–5507. 23150714 10.4049/jimmunol.1201413 PMC3518619

[bib48] Misumi I, Whitmire JK. B cell depletion curtails CD4+ T cell memory and reduces protection against disseminating virus infection. J Immunol 2014; 192: 1597–1608. 24453250 10.4049/jimmunol.1302661 PMC3925510

