# Supplementary material for: T-cell-dependent mechanisms promote Ebola VLP-induced antibody responses, but are dispensable for vaccine-mediated protection
Source: Emerg Microbes Infect. 2017 Jun 7;6(6):e46–. doi: 10.1038/emi.2017.31 (PMC5520308; doi:10.1038/emi.2017.31)
Supplement: Supplementary Figure S1 [file emi201731x1.pdf]

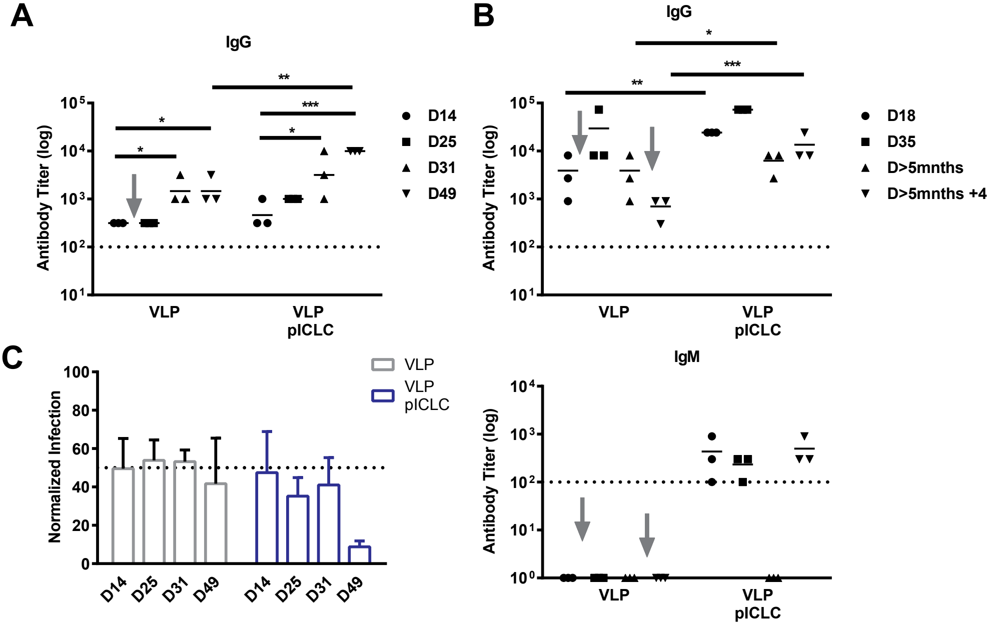

Supplementary Figure S1. PolyICLC enhances VLP-mediated acute and durable EBOV GP-specific humoral responses. Mice were vaccinated with VLP or VLP plus poly-ICLC at day 0, day 21, and day>5 months. A, B) EBOV $GP_{1,2}$-specific IgG titers measuring (A) acute-phase protection or (B) durable humoral responses. Sera were collected at the indicated time-points and EBOV $GP_{1,2}$-specific IgM and IgG responses were measured by ELISA. Titers were calculated by reciprocal end-point dilutions with background set at control absorbance + 0.2 O.D. (n=3). C) Neutralizing antibody titers during acute-phase protection. Sera sample neutralization were determined using recombinant vesicular stomatitis Indiana virus (rVSV) particles coexpressing EBOV $GP_{1,2}$ and enhanced green fluorescent protein (eGFP). Serum dilutions at 1:10 were incubated with rVSV and 5% v/v Hemo-lo guinea pig complement. rVSV/serum complexes were then incubated with 293F cells for 18-20 h. Percentage of infection, as determined by eGFP expression, was measured using fluorescence-activated cell sorting (FACS). Data were collected on a BD Canto II and LSR II. Neutralization was calculated by normalizing infection percentages to infections performed in the presence of control sera from non-vaccinated laboratory mice. Arrows indicate vaccination time points. Dashed lines within titer plots represent the limit of the assay. Dashed line within viral neutralization indicates level at which 50% reduction was achieved. ($^*p < 0.05$, $^{**}p < 0.005$, $^{***}p < 0.0005$).
