# Supplementary material for: T-cell-dependent mechanisms promote Ebola VLP-induced antibody responses, but are dispensable for vaccine-mediated protection
Source: Emerg Microbes Infect. 2017 Jun 7;6(6):e46–. doi: 10.1038/emi.2017.31 (PMC5520308; doi:10.1038/emi.2017.31)
Supplement: Supplementary Figure S2 [file emi201731x2.pdf]

A

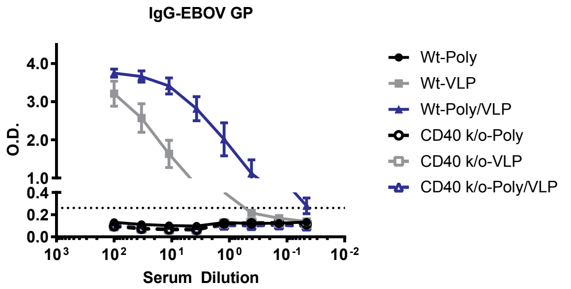

B

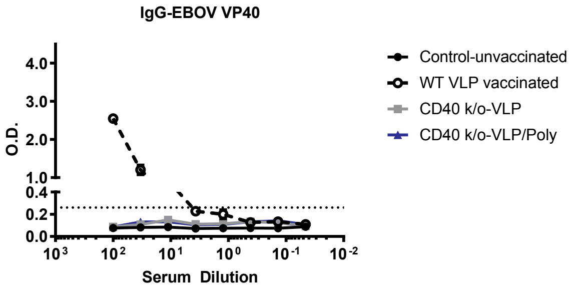

Supplementary Figure S2. CD40-deficient mice display a complete absence of VLP-induced antibody responses. Wild-type or CD40$^{-/-}$ mice were vaccinated with VLP with or without poly-ICLC at day 0 and day 21. Sera were collected at day 35 (day 14 post-boosting) and EBOV $GP_{1,2}$-specific or EBOV VP40-specific IgG responses were measured by ELISA. VP40 ELISA was performed under a modified protocol with overnight sera incubation to allow for measurement of low-affinity antibodies. Data represents absorbance at serum dilutions starting at 1:100. Dashed line denotes level of background of control absorbance +0.20 O.D. (n=10/group).
